# Laparoscopic Management of Seminoma in a Pediatric Patient: A Case Report

**DOI:** 10.7759/cureus.90453

**Published:** 2025-08-19

**Authors:** Katarzyna Polak, Julia Tarnowska, Marek Wolski

**Affiliations:** 1 Department of Paediatric Surgery, Medical University of Warsaw, Warsaw, POL

**Keywords:** laparoscopic surgery, management of seminoma, retroperitoneal lymph node dissection, seminoma in a pediatric patient, testicular tumor

## Abstract

Seminoma, a malignant neoplasm originating from germ cells, is a rare diagnosis in pediatric patients and seldom requires surgical intervention. In cases of retroperitoneal lymph node involvement, laparoscopic resection may reduce the invasiveness of the surgical intervention compared to traditional open surgery; however, it raises concerns about the oncological completeness of the procedure. We present a case report of an 11-year-old male patient who was admitted to the hospital for further diagnostic evaluation following abnormal imaging studies prompted by persistent headaches. A computed tomography (CT) scan of the abdomen revealed pathological mass in the left retroperitoneal space. A surgical biopsy of these lesions confirmed the diagnosis of seminoma. The patient was subsequently referred for chemotherapy, and after completing two cycles, a decision was made to proceed with laparoscopic resection of the retroperitoneal lymph nodes. The operation was performed without complications, and the patient continues to receive oncological follow-up. The selection of laparoscopic surgery enabled a reduction in the invasiveness of the surgical procedure while achieving a complete oncological resection of pathological lesions.

## Introduction

Seminoma is classified as a malignant tumor derived from germ cells. It most commonly occurs in males aged 15 to 35 years and is characterized by a high curability rate and a survival rate of 95-99% [1,2]. Testicular tumors in children are uncommon, accounting for 1-2% of all pediatric solid tumors. Metastases most often involve the retroperitoneal lymph nodes [3]. Testicular tumors in prepubescent boys exhibit a different histological distribution and lower malignancy compared to testicular tumors in adolescents and adults, which determines the recommended therapeutic approach. The EAU-ESPU guidelines from 2021 recommend partial orchiectomy as the primary treatment method for tumors with favorable preoperative ultrasound diagnoses [4]. Lymph node dissection in pediatric germ cell tumors, such as seminoma, plays a key role in evaluating disease spread and informing staging and treatment strategies. It is essential for detecting metastases and guiding effective management of the condition.

In cases involving retroperitoneal lymph node involvement, the laparoscopic method, by avoiding traditional open surgery (which involves a larger incision and direct exposure of the periaortic retroperitoneal space), offers the potential to reduce morbidity while leaving unresolved issues regarding the oncological completeness of the procedure.

Seminoma is an extremely rare diagnosis in the pediatric population and seldom requires surgical intervention for therapeutic purposes. Typically, after biopsy, chemotherapy is sufficient. This report presents a case of an 11-year-old boy with seminoma of the left testis and retroperitoneal lymph node involvement, who underwent laparoscopic resection of the lymph nodes and orchiectomy.

## Case presentation

An 11-year-old Caucasian male patient was admitted to the Oncology Clinic of the Pediatric Hospital at the Medical University of Warsaw for further diagnostic evaluation following abnormal imaging results. Ultrasound revealed tumor-like structures in the region of the left kidney, measuring 100x61x34 mm. The patient reported experiencing headaches for a year. He does not suffer from chronic illness and does not take medications chronically. A testicular ultrasound was also performed, showing abnormalities in the left testis (enlarged with a volume of 10 mL, heterogeneous with numerous hyperechoic reflections).

Headaches primarily occurred after exertion and during high temperatures, accompanied by vomiting. These vomiting episodes alleviated the headaches, prompting the patient to induce them frequently. The patient denied alarm symptoms (rapid weight loss, night sweats, low-grade fever, fever). Physical examination revealed asymmetry of the testes (left larger than right) and a mildly positive Goldflam sign on the left side. Laboratory tests revealed a decreased mean corpuscular volume (MCV) of 77.7 fL (normal range: 78-95 fL) and elevated levels of lactate dehydrogenase (LDH) at 450 U/L (normal range for 10- to 15-year-olds: 234-296 U/L).

**Figure 1:**
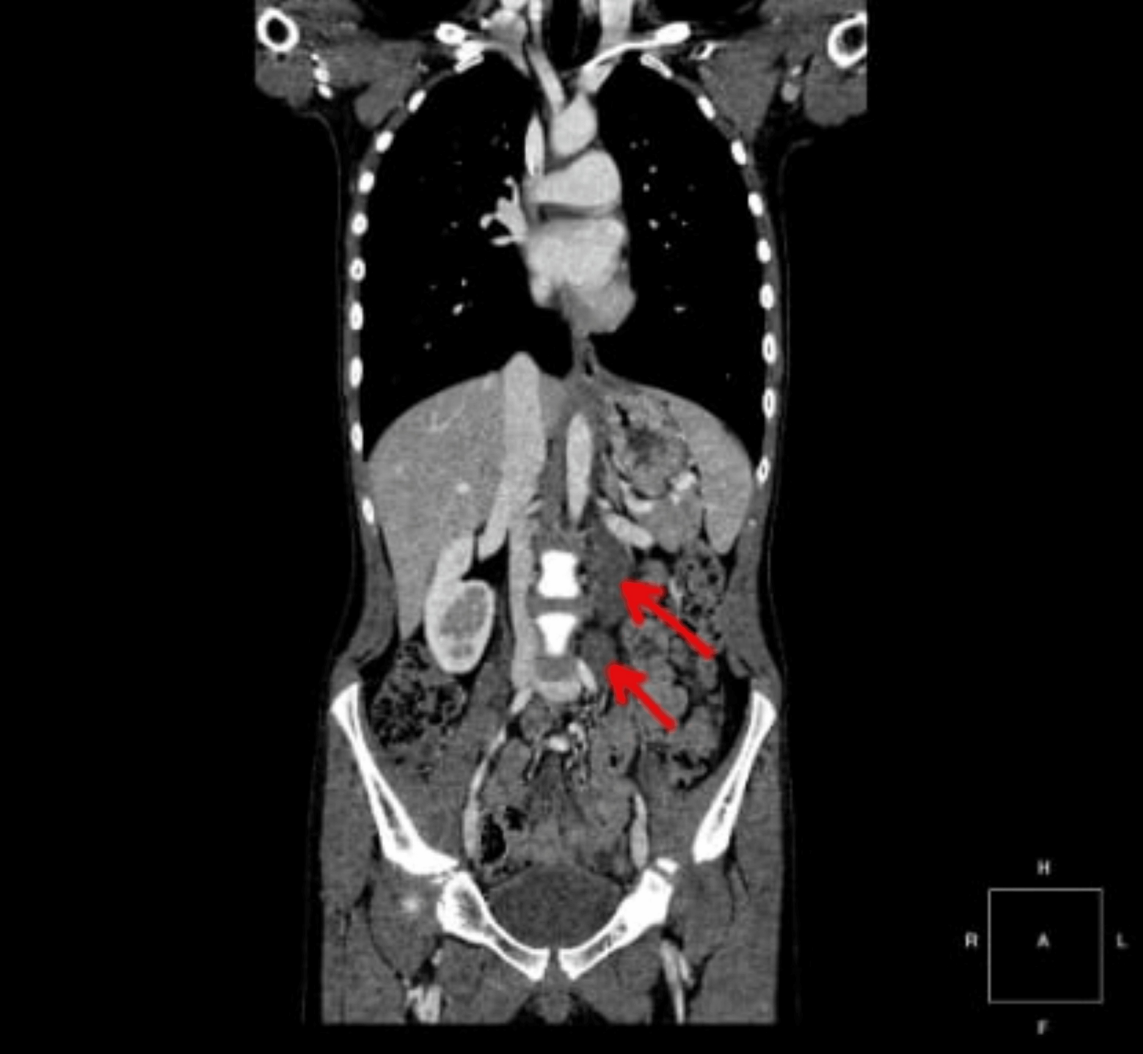
CT scan of the chest and abdomen showing enlarged lymph nodes in the left retroperitoneal space along the left psoas muscle (red arrows).

The next step involved expanding diagnostic imaging with a contrast-enhanced CT scan of the neck, chest, and abdomen, as illustrated in Figure 1. Pathological masses were described in the left retroperitoneal space between the aorta and left external iliac artery, left psoas muscle, left kidney, and bowel loops, measuring approximately 66 cc with slightly heterogeneous density affecting the left renal vein.

Burkitt lymphoma was suspected; however, levels of AFP (alpha-fetoprotein), HCG (human chorionic gonadotropin), and NSE (neuron-specific enolase) showed no significant elevation: AFP 1.84 ng/mL (normal range: <12 ng/mL), HCG <0.1 mIU/mL (normal range: <5 mIU/mL), and NSE 22.9 ng/mL (normal range: 12.5-25 ng/mL). A laparoscopic biopsy was performed. During the procedure, several tumors were visualized in the retroperitoneal area at the root of the small intestine on the left side. Samples were taken for histopathological examination. The diagnosis confirmed seminoma involving the left testis and retroperitoneal lymph nodes on the left side. Histopathological examination revealed positive markers in tumor cells: PLAP (placental alkaline phosphatase), c-kit (Proto-Oncogene c-kit), CALLA (common acute lymphoblastic leukemia antigen), and varied reaction for CK AE1-3 (Cytokeratin AE1-3), with negative reactions for EMA (epithelial membrane antigen) and CD30. Ki-67 showed 20-30% tumor cell proliferation.

The patient was classified into a low-risk group and scheduled for two cycles of VBP (vinblastine, bleomycin, and cisplatin) chemotherapy. After the first cycle of chemotherapy due to a lack of improvement regarding headaches, a repeat magnetic resonance imaging (MRI) was performed without abnormalities. Following chemotherapy completion, follow-up imaging (CT scan performed six weeks after completion of chemotherapy) demonstrated a reduction in lymph nodes on the left along the psoas muscle. The largest measured 16x22 mm compared to an initial size of 31x40 mm. The total volume of lymph node changes decreased from approximately 66 mL to about 16 mL. Laboratory tests indicated that LDH levels dropped to 205 U/L.

**Figure 2:**
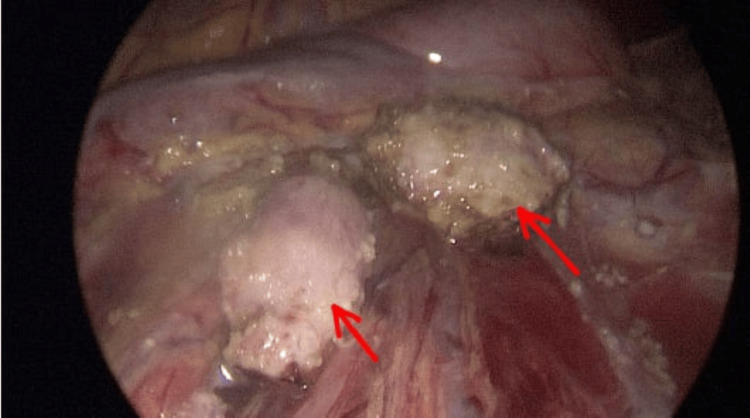
Laparoscopic view of nodular changes in retroperitoneal space (red arrows).

The next step involved laparoscopic resection of retroperitoneal lymph node changes (Figure 2) and left-sided orchiectomy via access through the left inguinal canal. Under direct vision, a 5 mm trocar was placed into the abdominal cavity. Three additional 5 mm trocars were inserted under optical guidance in the mid and lower abdomen. Enlarged lymph nodes were visualized beneath the renal vessels at the level of the aortic bifurcation. They were removed without complications. Subsequently, access to the left inguinal canal was opened. The left testis was repositioned near the superficial inguinal ring. Amputation was performed by clamping the spermatic cord at the level of the deep inguinal ring. Anesthesia lasted for 5 hours and 5 minutes; the surgery duration was 4 hours and 15 minutes with minimal blood loss. The perioperative period was uncomplicated. The patient was fed the day after the surgery, the drain was removed on the second day, and the patient was discharged home on the third day.

The excised material was sent for histopathological examination. Macroscopically, on cross-sections, a poorly defined, white, solid, homogeneous tumor measuring 4.1x2.2x2.1 cm was observed within the testis, occupying approximately 80% of its volume without apparent infiltration into surrounding structures. During the procedure, three lymph nodes were removed, which, upon histopathological examination, were described as cohesive tissue fragments containing no viable cells. Seminoma at stage IIIB was diagnosed.

Postoperatively, follow-up CT scans did not reveal any presence of neoplastic tissue. Air was noted within the scrotum. Routine follow-up abdominal ultrasound examinations and scrotal ultrasounds performed after three weeks, 4.5 weeks, and 4.5 months showed no signs of tumor recurrence or increased fluid accumulation. The Broviac catheter was removed after 16 weeks. The patient remains in good general condition under oncological care.

## Discussion

Laparoscopic retroperitoneal lymph node dissection (L-RPLND) is increasingly recognized as an effective therapeutic method for patients with non-seminomatous germ cell tumors and serves as a viable alternative to open retroperitoneal lymph node dissection (O-RPLND) [5]. It significantly reduces intraoperative blood loss (155 mL for L-RPLND vs. 700 mL for O-RPLND) while achieving comparable oncological efficacy with improved recovery parameters [1,6,7].

An analysis comparing open versus laparoscopic lymphadenectomy in 30 patients with germ cell tumors demonstrated substantial benefits from L-RPLND. The laparoscopic approach significantly reduced intraoperative blood loss (an average of 165 mL for L-RPLND compared to 403 mL for O-RPLND; p<0.001) and shortened operative time (an average of 222 minutes for L-RPLND vs. 453 minutes for O-RPLND; p<0.001). Postoperative recovery parameters were markedly better in the L-RPLND group; patients undergoing laparoscopic procedures were discharged from the hospital on average after three days compared to 11 days for open procedures [8].

Various sources report significantly higher complication rates in O-RPLND compared to L-RPLND (86.2% vs. 15%, p<0.001) [9] or show no significant difference between both surgical methods (L-RPLND: 18% vs. O-RPLND: 17%) [10].

The literature documents successful applications of laparoscopic treatment for seminomas in adult populations. Reported surgeries exhibit a maximum blood loss of up to 300 mL with operative times ranging from 150 to 210 minutes and hospital discharge within 48 hours while maintaining comparable oncological effects [2].

While concerns regarding oncological completeness persist with laparoscopic techniques for tumor resection, this is not a critical argument regarding seminoma histology. The principal objective of the surgical intervention in this context is to achieve maximal cytoreduction of the tumor mass while preserving the anatomical and functional integrity of adjacent critical structures. The case we described, along with other literature evidence, suggests that laparoscopic techniques can maintain equivalent oncological efficacy compared to open approaches in both adult and pediatric populations.

## Conclusions

The presented case report highlights the potential of laparoscopic methods for removing lymph nodes affected by seminoma morphology tumors. This approach minimizes blood loss during surgery and facilitates rapid recovery. Laparoscopic lymphadenectomy techniques provide opportunities for complete resection of neoplastic changes while reducing surgical extent when performed by experienced operators; however, further monitoring of long-term treatment outcomes is necessary. The choice of laparoscopic retroperitoneal lymphadenectomy offers numerous advantages over traditional open techniques. It significantly reduces intraoperative blood loss, shortens operative time, accelerates recovery, and facilitates complete resection of neoplastic.
